# Machine learning in the development and application of patient-reported outcome measures (PROMs) for surgical patients: a systematic review

**DOI:** 10.1186/s41687-026-00992-8

**Published:** 2026-01-14

**Authors:** Tariq Alanezi, Ben Li, Leen Al-Omran, Lina Alshabanah, Nawaf K. Alkhayal, Meena Verma, Husam Alrumaih, Mohamad A. Hussain, Muhammad Mamdani, Mohammed Al-Omran

**Affiliations:** 1 Division of Vascular Surgery, Department of Surgery, University of Toronto, Toronto, ON Canada; 2 Department of Surgery, University of Toronto, Toronto, ON Canada; 3 Division of Vascular Surgery, St Michael’s Hospital, Unity Health Toronto, 30 Bond Street, Suite 7-074, Bond Wing, Toronto, ON M5B 1W8 Canada; 4 Institute of Medical Science, University of Toronto, Toronto, ON Canada; 5 Temerty Centre for Artificial Intelligence Research and Education in Medicine (T-CAIREM), University of Toronto, Toronto, ON Canada; 6 College of Medicine, Alfaisal University, Riyadh, Saudi Arabia; 7 School of Medicine, Royal College of Surgeons in Ireland, Dublin, Ireland; 8 Department of Orthopedics, King Faisal Specialist Hospital and Research Center, Riyadh, Saudi Arabia; 9 Harvard Medical School, Boston, MA USA; 10 Division of Vascular and Endovascular Surgery and the Center for Surgery and Public Health, Department of Surgery, Brigham and Women’s Hospital, Boston, MA USA; 11 Data Science & Advanced Analytics, Unity Health Toronto, University of Toronto, Toronto, ON Canada; 12 Institute of Health Policy, Management and Evaluation, University of Toronto, Toronto, ON Canada; 13 Institute for Clinical Evaluative Sciences, University of Toronto, Toronto, ON Canada; 14 Li Ka Shing Knowledge Institute, St. Michael’s Hospital, Unity Health Toronto, Toronto, ON Canada; 15 Leslie Dan Faculty of Pharmacy, University of Toronto, Toronto, ON Canada; 16 Department of Surgery, King Faisal Specialist Hospital and Research Center, Riyadh, Saudi Arabia

**Keywords:** Artificial intelligence, Machine learning, Patient-reported outcome measures, Surgical care, Post-surgical outcomes

## Abstract

**Background:**

Artificial intelligence (AI) and machine learning (ML) are increasingly integrated into healthcare, offering potential advancements in patient-reported outcome measures (PROMs) for surgical populations. Improved PROMs can enhance patient-centered care by accurately capturing patient experiences with minimal burden.

**Objective:**

In the context of surgery, where recovery trajectories vary widely, this study aims to systematically review the use of AI and ML in the development, application, and prediction capabilities of PROMs in surgical populations, with a focus on psychometric properties and the predictive accuracy of post-surgical outcomes.

**Methods:**

A comprehensive search of the PubMed database was conducted from inception until August 2024. Studies were included if they utilized AI or ML in the development, application, or predicting PROMs for surgical patients. Methodological quality was assessed using COSMIN and PROBAST tools, depending on study design. A qualitative synthesis of findings was performed.

**Results:**

Twenty-two studies met the inclusion criteria, with 19 rated as high quality. Six studies focused on developing computer adaptive tests (CAT) PROMs, seven on evaluating psychometric properties, and five on ML for post-surgical outcome prediction. CAT PROMs showed comparable measurement accuracy to traditional PROMs, good to excellent construct validity, and significantly reduced patient burden by reducing the length of questionnaires. ML algorithms, such as logistic regression, random forests, extreme gradient boosting, and neural networks, achieved similar predictive accuracy for post-surgical outcomes, with no single model demonstrating consistent superiority.

**Conclusions:**

AI and ML have the potential to improve PROM utilization in surgical care by enhancing efficiency and personalization while maintaining data quality. Clinicians can use AI-driven PROMs to reduce patient burden and integrate ML models for accurate post-surgical outcome prediction, thereby optimizing patient-centered care.

**Supplementary Information:**

The online version contains supplementary material available at 10.1186/s41687-026-00992-8.

## Introduction

The integration of Artificial Intelligence (AI) and its subfield Machine Learning (ML) [1], into healthcare and research is primed to significantly transform current practices. One of the notable areas of impact is the development and application of patient-reported outcome measures (PROMs) [2]. PROMs capture patients’ perspectives on their health, treatment satisfaction, and overall quality of life, making them essential for evaluating intervention outcomes. Traditionally, PROMs have relied on standardized questionnaires that, while useful, can be limited by their rigidity, length, and potential to place a significant burden on patients. The rise of AI has introduced a new paradigm for PROMs, particularly through the development of computer adaptive tests (CATs) [3–5]. CATs leverage AI algorithms to dynamically tailor questionnaires to each patient, selecting subsequent questions based on previous responses from an extensive item bank of potential queries. In theory, this adaptive approach reduces the number of questions needed to obtain precise measurements while enhancing the relevance of questions to individual patients, without compromising data quality.

AI-supported CAT PROMs are especially relevant in surgical populations due to the complex and dynamic nature of surgical recovery. Outcomes in surgical patients can vary widely based on the type of surgery, patient-specific factors, and postoperative care [6, 7]. AI and ML technologies offer the potential to develop more personalized and adaptive PROMs that can capture this variability, while minimizing patient burden. This adaptability makes them particularly valuable for tracking the nuanced recovery trajectories seen in surgical patients.

Another promising area of application of ML algorithms is in predicting patient-reported surgical outcomes. Algorithms such as logistic regression, random forests (RF), and neural networks can detect complex data patterns, enabling better preoperative planning, risk stratification, and informed decision-making [8]. These models are also capable of continuous learning with improved prediction accuracy as new data becomes available, ultimately enhancing both patient care and surgical success rates [9].

In this systematic review, we aim to examine the role of AI and ML in the development and implementation of PROMs within surgical populations. We focus on AI-supported CATs for PROM development and administration, and ML algorithms for predicting surgical outcomes using PROM and related data. Given this strategy, we seek to explore how these technologies enhance the assessment of patient outcomes, optimize the length and content of PROM tools, and contribute to more patient-centered care in surgical settings. Specifically, we will assess how AI and ML have influenced measurement characteristics (e.g., reproducibility, validity, responsiveness) and user experience (e.g., reduction of questionnaire burden) in surgical populations, as well as how ML algorithms have been utilized for PROM-based outcome prediction models.

## Methods

### Search strategy

A systematic review of PubMed-indexed literature was performed on August 12, 2024, following the Preferred Reporting Items for a Systematic Review and Meta-analysis (PRISMA) [10] checklist. No restrictions were placed on publication year or language, although the search timeframe was from inception to August 2024. A search syntax covering four domains was developed: 1) PROMs, 2) AI/ML, 3) measurement characteristics and patient experience, and 4) surgical populations. For PROMs, key terms included ‘patient-reported outcome’, ‘self-report’, ‘questionnaire’, ‘survey’, and ‘Likert scale’. The AI/ML domain utilized terms such as ‘artificial intelligence’, ‘machine learning’, ‘algorithm’, ‘computer-adaptive’, and ‘modifiable’. The third domain, measurement characteristics and user experience, adapted the COnsensus-based Standards for the selection of health Measurement INstruments (COSMIN) PubMed search filter for measurement properties [11], excluding terms unrelated to PROMs (e.g., ‘inter-rater’ or ‘inter-observer’) and adding user experience terms like ‘acceptability’ and ‘applicability’. For surgical populations, key terms included ‘surgery’, ‘surgical intervention’, and suffixes such as ‘-scopy’ and ‘-tomy’. The full search syntax is available in the Supplementary file.

### Eligibility criteria

Studies published before August 12, 2024, were included if they presented original prospective or retrospective analyses on individuals indicated for, or who had undergone, surgery for any health-related condition, and if they examined the role of AI/ML in developing, applying, or predicting PROMs. Papers that lacked primary analyses (e.g., editorials, theoretical papers, or reviews), case reports and case-series, or those focused on non-PROM measurement instruments (e.g., observational tests) were excluded. During the screening process, we examined the reference lists of relevant systematic and narrative reviews to identify any additional eligible primary studies that might not have been captured by our search strategy.

### Study selection

Two independent reviewers screened the titles and abstracts of all unique citations. For studies meeting the inclusion criteria or where eligibility was uncertain based on title and abstract alone, the full text was reviewed. Discrepancies between reviewers were resolved through discussion. If consensus could not be reached, a third reviewer was consulted.

### Data extraction

From each included study, data on bibliographical information, study design, purpose, population characteristics (e.g., type of surgery), sample size, and all relevant findings on AI/ML use in developing, applying, or predicting PROMs were extracted. The study protocol was registered in the International Prospective Register of Systematic Reviews (PROSPERO) under the registration number CRD42024591696.

### Methodological quality of included studies

The methodological quality of the included studies was assessed by evaluating the risk of bias. Studies focused on the measurement properties of AI-informed PROMs (e.g., CATs) or the development of new PROMs using AI were assessed using the COSMIN risk of bias tool [12]. In this tool, all information relating to how measurement properties are assessed is rated on a 4-point scale, according to well-defined criteria (1 = very good, 2 = adequate, 3 = doubtful, 4 = inadequate). Then, as advised by COSMIN methodology, the overall quality was determined by the study’s worst-performing aspect. If the lowest score was 1 or 2, the study was rated as high quality. A score of 3 or 4 indicated low quality.

The COSMIN tool was not appropriate to assess all studies included in this systematic review, as it was specifically developed to evaluate studies on the quality of measurement tools. Therefore, for studies using ML algorithms to predict PROM outcomes, methodological quality in terms of risk of bias and applicability was evaluated using the PROBAST checklist [13]. This checklist rates the risk of bias for four design choices in prediction modelling studies (participants, choice of predictors, choice of outcome, analysis) and applicability for three elements of the study design (participants, predictors, and outcome). Risk of bias was considered low if a maximum of one item was rated as “doubtful” and all others as “low risk.” If more than one item was rated “doubtful” or any item was rated “high risk,” the study was classified as high risk. Applicability was rated as good if no more than one item was rated “doubtful,” and poor if more than one item was “doubtful” or any was rated “high concern.” A study was deemed high quality if both risk of bias and applicability were rated favorably. If either risk of bias was high or applicability was poor, the study was considered low quality. The rationale for using two different risk of bias assessment tools stems from the specific focus of each. The COSMIN tool is designed to assess studies that evaluate the quality of measurement tools, which were applied to the majority of the papers included. However, for the ML modeling studies, COSMIN was not suitable as it would not provide a valid assessment of methodological quality. Therefore, the more appropriate PROBAST tool was employed to accurately assess the risk of bias in these modeling studies.

### Data analysis

A qualitative analysis of the results from the included studies was conducted. The significant heterogeneity of the PROM tools utilized, along with the wide range of other PROMs used for comparison, made it challenging to standardize the data across the studies. As a result, data pooling was not feasible, and instead, individual study outcomes were assessed qualitatively to capture the nuances of their findings.

## Results

Out of 6463 records identified in the systematic search, 31 studies were selected for full-text review. After excluding 9 studies that did not meet the inclusion criteria, 22 studies were ultimately included in the systematic review. Of these, 6 studies focused on the development of CAT PROMs, 7 evaluated the psychometric properties (which indicates the measurement tool quality relating to its reproducibility, validity, and responsiveness) of CAT PROMs, and 3 explored the application of CAT PROMs in specific surgical populations. Five studies used ML to model PROM outcomes, while 1 study employed ML to develop a short-form (non-CAT) version of an existing PROM. PRISMA flow chart summarizing article screening can be found in Supplementary Fig. 1. A summary of all 22 included studies is provided in Table 1.

**Table 1:** Descriptive information of the included studies

Reference	Country	Population / Surgical specialty	Design	*N*	Type of study	PROM	Domain
Giesinger et al., 2013	Austria	TKA and THA (Orthopedic surgery)	Cross-sectional > 1 year post-surgery	580	CAT PROM development	FJS	Joint awareness
Hafner et al., 2023	USA	Unilateral lower limb amputation due to trauma or vascular complications (Physical Medicine & Rehabilitation)	Cross-sectional; post-surgery	1091	CAT PROM development	PLUS-M	Mobility
Harrison et al., 2023	International (multicenter: Canada, UK, USA, others)	Cleft lip, palate, and/or alveolus (Plastic surgery)	Cross-sectional, simulation, qualitative	2434 / 536 / 6	CAT PROM development	CLEFT-Q	Appearance of the face, teeth, and jaws; speech function and speech distress; school, social, and psychological function
Guattery et al., 2018	USA	Not specified (Orthopedic surgery)	Cross-sectional, pre-surgery	76,574	CAT psychometric assessment	PROMIS-PF; PROMIS-Depression	Physical function; depression
Banerjee et al., 2020	USA	THA (Orthopedic surgery)	Cross-sectional, simulation	354 + 1547	CAT PROM development	HOOS and HOOS-Joint Replacement	Pain, symptoms, stiffness, physical function and QoL
Anthony et al., 2017(1)	USA	Rotator cuff pathology (Orthopedic surgery)	Cross-sectional, pre-surgery	82	CAT psychometric assessment	PROMIS-UE, PROMIS-PF	Upper extremity pathology (PROMIS-UE); physical function and musculoskeletal health (PROMIS-PF)
Young Afat et al., 2019	USA	Reconstructive breast surgery (Plastic surgery)	Cross-sectional, post-surgery simulation	5000	CAT PROM development	BREAST-Q	HRQoL, satisfaction with surgery outcome
Lötsch et al., 2018	Germany/Finland	Breast cancer surgery (Oncological surgery)	Longitudinal, post-surgery	1000	Short-form PROM development using machine learning	Short form combining items from BDI and STAI	Psychological factors predicting pain persistence after surgery
Campagner et al., 2024	Italy	TKA and THA (Orthopedic surgery)	Longitudinal, pre- and post-surgery	899	PROM application using machine learning	SF-12 physical score	Physical function
Huber et al., 2019	Germany	TKA and THA (Orthopedic surgery)	Longitudinal, pre- and post-surgery	130,945	PROM application using machine learning	EQ-5D-3 L VAS, Oxford Hip and Knee Score (Q score)	EQ-5D-3 L VAS: mobility, self-care, activities of daily life, pain, anxiety/depression; Q Score: disease-specific HRQoL
Zhou et al., 2023	Australia	TKA (Orthopedic surgery)	Longitudinal, pre- and post-surgery	3755	PROM application using machine learning	VR-12	HRQoL
Kumar et al., 2020	USA	TSA (Orthopedic surgery)	Longitudinal, pre- and post-surgery	4782	PROM application using machine learning	ASES, VAS pain	Activities of daily living, pain
Harris et al., 2021	USA	TKA (Orthopedic surgery)	Longitudinal, pre- and post-surgery	587	PROM application using machine learning	KOOS joint replacement	Pain, symptoms, activities of daily life, recreation, QoL
Harrison et al., 2022	UK	Hand surgery (Orthopedic / Plastic surgery)	Cross-sectiona, pre- and post-surgery.	507	CAT PROM development	DASH	Motor function, sensory symptoms
Hajewski et al., 2022	USA	Surgery for shoulder instability (Orthopedic surgery)	Longitudinal, pre- and post-surgery	72	CAT psychometric assessment	PROMIS-UE, PROMIS-PF	Upper extremity pathology (PROMIS-UE); physical function and musculoskeletal health (PROMIS-PF)
Rojas et al., 2019	USA	Elbow surgery (Orthopedic surgery)	Cross-sectional, pre-surgery	76	CAT psychometric assessment	PROMIS-UE, PROMIS-PF	Upper extremity pathology (PROMIS-UE); physical function and musculoskeletal health (PROMIS-PF)
Dowdle et al., 2017	USA	TSA (Orthopedic surgery)	Cross-sectional, pre-surgery	53	CAT psychometric assessment	PROMIS-UE, PROMIS-PF	Upper extremity pathology (PROMIS-UE); physical function and musculoskeletal health (PROMIS-PF)
Day et al., 2021	USA	ACL reconstruction (Orthopedic surgery)	Cross-sectional, pre-surgery	1126	CAT psychometric assessment	PROMIS Mobility, PROMIS-PI	Mobility (PROMIS Mobility); interference of pain with daily life (PROMIS-PI)
Anthony et al., 2017(2)	USA	Surgery for shoulder instability (Orthopedic surgery)	Cross-sectional, pre-surgery	70	CAT psychometric assessment	PROMIS-UE, PROMIS-PF	Upper extremity pathology (PROMIS-UE); physical function and musculoskeletal health (PROMIS-PF)
Tenan et al., 2021(1)	USA	Shoulder surgery (Orthopedic surgery)	Longitudinal, pre- and post-surgery	909	CAT PROM application	PROMIS-PF, PROMIS-PI	Physical function and musculoskeletal health (PROMIS-PF); interference of pain with daily life (PROMIS-PI)
Tenan et al., 2021(2)	USA	Knee surgery (Orthopedic surgery)	Longitudinal, pre- and post-surgery	1011	CAT PROM application	PROMIS-PF, PROMIS-PI	Physical function and musculoskeletal health (PROMIS-PF); interference of pain with daily life (PROMIS-PI)
Hoogendam et al., 2022	Netherlands	Carpal tunnel release from the Hand and wrist cohort (Plastic surgery)	Longitudinal, pre- and post-surgery	2119 / 397	PROM application using machine learning	BCTQ; VAS pain, function, and satisfaction	Symptom severity, functional status (BCTQ); pain, function, satisfaction with surgery (VAS)

Use of/in the N column denotes separate sample sizes for different parts of the study, while use of + denotes combined samples within the same part of the study. Abbreviations: ACL = anterior cruciate ligament, ASES = American Shoulder and Elbow Surgery, BDI = Beck’s Depression Inventory, BCTQ = Boston carpal tunnel questionnaire, CAT = computer-adaptive test, DASH = Disabilities of the Arm, Shoulder and hand, FJS = Forgotten Joint Score, HOOS = Hip Disability and Osteoarthritis Outcome Score, HRQoL = health-related quality of life, KOOS = Knee Injury and Osteoarthritis Outcome Score, PLUS-M = Prosthetic Limb Users Survey of Mobility, PROM = patient-reported outcome measure, PROMIS = patient-reported outcome measure information system, PROMIS-PF = PROMIS physical function, PROMIS-PI = PROMIS pain interference, PROMIS-UE = PROMIS upper extremity, QoL = quality of life, SF-12 = Short-Form 12, STAI = Spielberger’s State-Trait Anxiety Inventory, THA = total hip arthroplasty, TKA = total knee arthroplasty, TSA = total shoulder arthroplasty, VAS = visual analogue scale, VR-12 = Veteran’s RAND-12

### Methodological quality

Sixteen studies were assessed using the COSMIN risk of bias tool [12] (Table 2) For details on each implemented PROM tool, please refer to Table 1. Of these, 13 were rated as high quality and 3 as low quality, the latter due to insufficient sample sizes. The remaining six studies were assessed using the PROBAST tool [13] and were all rated as high quality (Table 3).

**Table 2:** Assessment of the methodological quality of the included publications that develop PROMS using the COSMIN risk of bias tool

Publication	Risk of bias assessment										
	PROM design	Content validity	Structural validity	Internal consistency	Cross-cultural validity / invariance	Reliability	Measurement error	Criterion validity	Construct validity (hypothesis testing)	Responsiveness	Overall quality
Giessinger et al., 2013	1	x	1	x	x	x	1	x	2	x	High
Hafner et al., 2023	1	1	1	x	1	1	1	x	1	x	High
Harrison et al., 2023	1	x	2	x	x	x	1	x	1	x	High
Guattery et al., 2018	X	x	x	x	1	x	1	x	X	x	High
Banerjee et al., 2020	X	x	x	x	x	x	1	x	1	x	High
Anthony et al., 2017(1)	X	x	x	x	x	x	x	x	2	x	High
Young Afat et al., 2019	1	x	1	2	x	x	1	x	1	x	High
Lötsch et al., 2018	2	2	1	x	x	x	x	1	1	x	High
Harrison et al., 2022	2	1	1	x	x	x	1	x	X	x	High
Hajewski et al., 2022	X	x	x	x	x	x	x	x	2	2	High
Rojas et al., 2019	X	x	x	x	x	x	x	x	3	x	Low
Dowdle et al., 2017	X	x	x	x	x	x	x	x	3	x	Low
Day et al., 2021	X	x	x	x	x	x	x	x	2	x	High
Anthony et al., 2017(2)	X	x	x	x	x	x	x	x	3	x	Low
Tenan et al., 2021(1)	X	x	x	x	x	x	x	x	2	x	High
Tenan et al., 2021(2)	X	x	x	x	x	x	x	x	2	x	High

Risk of bias assessment values: 1 = very good; 2 = adequate, 3 = doubtful, 4 = inadequate, x = not assessed (item not present in publication). Overall quality: high if all assessed domains score 1 or 2; Low if at least one of the assessed domains scores 3 or 4

**Table 3:** Assessment of the methodological quality of publications using machine learning for PROM prediction modelling using the PROBAST tool

Publication	Risk of Bias				Applicability			Overall		
	Participants	Predictors	Outcome	Analysis	Participants	Predictors	Outcomes	Risk of Bias	Applicability	Quality
Campagner et al., 2024	?	+	+	+	+	+	+	+	+	High
Huber et al., 2019	+	+	+	+	+	+	+	+	+	High
Zhou et al., 2023	+	+	+	+	+	+	+	+	+	High
Kumar et al., 2020	+	+	+	?	+	+	+	+	+	High
Harris et al., 2021	+	+	+	+	+	+	+	+	+	High
Hoogendam et al., 2022	+	+	+	+	+	+	+	+	+	High

+ = low risk of bias / low concern on applicability; ? = unclear risk of bias / unclear concern on applicability; - = high risk of bias / high concern on applicability

### Psychometric quality of CAT PROMs

The majority of evidence from the included studies focused on the psychometric quality of CAT PROMs. All CATs were designed to measure a single health domain, with three studies assessing unidimensionality (i.e., whether the CAT PROM did indeed measure a single health domain) [14–16]. Using measures such as the root mean square error of approximation (RMSEA) and variance-accounted-for (R²) from a one-factor principal component analysis (PCA), CAT unidimensionality was rated as good to excellent in the included studies. Reported RMSEA values were 0.06 for the Forgotten Joint Score (FJS) CAT in a knee arthroplasty population and 0.10 for a hip arthroplasty population [14]; 0.057 for the Prosthetic Limb Users Survey - Mobility scale (PLUS-M) [15]; and 0.05 for the CLEFT-Q [16]. All of these values meet the accepted threshold of 0.10 or below, indicating good unidimensionality [17]. For the FJS, the R² of the one-factor PCA solution was 0.714 [14], indicating good performance.

Measurement error was examined in five studies [14, 16, 18–20]. The standard error, which is dependent on the number of administered items/questions in a CAT, met the accuracy threshold for group-based research (standard error < 0.55) [21] for all CATs that administered more than three items. However, higher measurement error was observed when the CAT length was reduced to one or two items, with standard error > 0.55 in all reported cases.

Floor and ceiling effects were reported in seven studies [14–16, 18−20, 22]. Although more patients scored the highest possible (ceiling effect) or lowest possible (floor effect) score in the CAT PROMs compared to full-length non-CAT PROMs, these elevated floor and ceiling effects remained within acceptable limits (< 15% of observations) [23] in all but one case. Hajewski et al. [24] reported high ceiling effects for the Patient-Reported Outcome Measure Information System Upper Extremity item bank (PROMIS-UE) in a post-surgical shoulder instability population, with 68.1% and 67.0% of patients obtaining the maximum possible score for the PROMIS-UE at 6 months and 2 years post-surgery, respectively.

Construct validity was the most frequently assessed psychometric property, by correlating CAT PROM scores with other PROMs. Table 4 summarizes the construct validity findings from 10 studies. Three studies [16, 19, 20] compared simulated CAT PROM scores with the original full PROM scores, demonstrating near-perfect correlation (*r* ≥ 0.98) and agreement (ICC ≥ 0.98). Other studies correlated CAT PROM scores with PROMs measuring the same health domains, with high expected correlations, or different health domains, where correlation was expected to be lower (Table 4). In all cases, construct validity was rated as good to excellent; correlations between the CAT PROM and other PROMs measuring the same health domain all showed *r* > 0.6 (indicating good construct validity, with many exceeding *r* > 0.8 (indicating excellent construct validity). Correlations between CAT PROMs and other PROMs measuring different health domains were lower than *r* = 0.6, showing divergent construct validity [25, 26]. Other psychometric properties were less frequently evaluated. Hajewski et al. [24] examined the responsiveness of two PROMIS CATs: the PROMIS-UE and the PROMIS-PF (physical function scale). Both demonstrated sensitivity to change with medium (ES > 0.5) to large (ES > 0.8) effect sizes. For the PROMIS-UE, effect sizes were 1.05 at 6 weeks and 1.09 at 6 months post-surgery; for the PROMIS-PF, they were 0.94 and 0.76 at the same time points.

**Table 4:** Construct validity of CAT PROMs

Publication	Design	CAT PROM name	Construct validity (values of correlations with other PROMs)
Hafner et al., 2023	Primary data collection	PLUS-M	PEQ-MS 0.864 PROMIS-PF 0.842 PROMIS-PI − 0.466 PROMIS-F − 0.501 PROMIS-D − 0.446
Harrison et al., 2023	Simulation using existing data	CLEFT-Q	Full CLEFT-Q: >0.97
Banerjee et al., 2020	Simulation using existing data	HOOS HOOS-JR	Full HOOS: 0.99 Full HOOS-JR: 0.99
Anthony et al., 2017(1)	Primary data collection	PROMIS-UE PROMIS-PF	ASES 0.77 WORC 0.73 EQ-5D 0.73 SF-36 PF 0.66 SF-36 GH 0.30 ASES 0.55 WORC 0.61 EQ-5D 0.65 SF-36 PF 0.77 SF-36 GH 0.50
Young Afat et al., 2019	Simulation using existing data	BREAST-Q Satisfaction	Full BREAST-Q satisfaction 0.89–0.98
Hajewski et al., 2022	Primary data collection	PROMIS – UE PROMIS – PF	ASES 0.73 WOSI 0.62 SF-36 PF 0.68 ASES 0.64 WOSI 0.64 SF-36 PF 0.62
Rojas et al., 2019	Primary data collection	PROMIS – UE PROMIS – PF	EQ-5D 0.62 SF-36 subscales EWB 0.15 Vitality 0.32 SF 0.20 Pain 0.53 GH 0.34 EQ-5D 0.74 SF-36 subscales EWB 0.19 Vitality 0.36 SF 0.36 Pain 0.62 GH 0.24
Dowdle et al. 2017	Primary data collection	PROMIS – UE PROMIS – PF	ASES 0.55 EQ-5D 0.48 WOOS 0.34 SF-36 PF 0.53 ASES 0.64 EQ-5D 0.64 WOOS 0.51 SF-36 PF 0.81
Day et al., 2021	Primary data collection	PROMIS Mobility PROMIS-PI	IKDC 0.81 SANE 0.46 IKDC − 0.75 SANE − 0.40
Anthony et al., 2017(2)	Primary data collection	PROMIS – UE PROMIS – PF CAT	ASES 0.71 WOSI 0.63 EQ-5D 0.66 SF-36 PF 0.78 ASES 0.67 WOSI 0.64 SF-36 PF 0.72

Abbreviations: ASES = American Shoulder and Elbow Surgery, CAT = computer-adaptive test, EQ-5D = Euro Quality of Life 5 Dimensions, HOOS = Hip Disability and Osteoarthritis Outcome Score, HOOS-JR = HOOS for joint replacement, IKDC = International Knee Documentation Committee, PLUS-M = Prosthetic Limb Users Survey of Mobility, PEQ-MS = Prosthesis Evaluation Questionnaire-Mobility Section, PROM = patient-reported outcome measure, PROMIS = patient-reported outcome measure information system, PROMIS-D = PROMIS disability, PROMIS-F = PROMIS fatigue, PROMIS-PF = PROMIS physical function, PROMIS-PI = PROMIS pain interference, PROMIS-UE = PROMIS upper extremity, SANE = single assessment numeric evaluation, SF = Short-Form, VAS = visual analogue scale, WOSI = Western Ontario Shoulder Instability Index. Notes: All values are Pearson correlations (r). Positive r indicates both instruments’ scores in the same direction; negative r indicates opposite scoring directions (e.g., higher pain vs. higher function). Magnitude guidelines (rule-of-thumb): ~0.10 small, ~ 0.30 moderate, ~ 0.50 + large; |r| close to 1.0 is very high. Correlation reflects association, not agreement. Where authors reported thresholds a priori (e.g., ≥ 0.5 for convergent; ≤0.3 for divergent), we evaluated “hypothesis met” using those thresholds; otherwise, conventional thresholds above were used

### Use of CAT PROMs to construct other PROM scores

Two studies [27, 28] successfully utilized data from the PROMIS-PF and PROMIS-Pain Interference (PROMIS-PI) CATs to construct scores for other well-established, diagnosis-specific PROMs. Both studies utilized general additive mixed models (GAMMs), a specific type of regression analysis, to map PROMIS-PF and PROMIS-PI data to the other diagnosis-specific PROM.

In one study [28], PROMIS data approximated the American Shoulder and Elbow Surgeons (ASES) score to within 13 points. The result is 7 points below the minimal clinically important difference (MCID), referred to individual patient change; specifically, the MCID for the ASES was 21.7, and the best ASES index MCID was 19.4. Therefore, the ASES score estimated from PROMIS-PF and/or PROMIS-PI data was within the error margin of the original ASES score (7 points below the MCID of 21.7), indicating close agreement between the modeled and original scores. Similarly, in the other study [27], the International Knee Documentation Committee (IKDC) score was modeled with higher accuracy than the MCID. The Gaussian and beta distribution algorithms were validated for predicting IKDC scores, yielding Pearson correlations of 0.84–0.86, R² values of 0.71–0.74, and RMSE of 9.3–10.0. Interestingly, in both studies, the mean prediction error was smaller than the respective MCID thresholds, meaning that differences between the predicted and actual scores were not clinically significant.

### Use of machine learning in PROM modelling

Seven studies employed ML techniques to model PROM outcomes. Five studies [29–33] used ML algorithms to predict postoperative PROM improvement beyond the MCID. In all five studies, pre-operative input data included PROM scores and other patient-reported data (e.g., symptom severity, lifestyle characteristics) as well as demographic information (e.g., age, gender). These input parameters were then used to predict PROM scores at various time points post-operatively, varying from 3 months to 2 years post-op. Across these studies, various ML algorithms, including logistic regression, extreme gradient boosting (XGB), RFs, multi-step elastic net, linear models, and Wide & Deep networks, were found to accurately predict PROM improvement above the MCID following surgery.

Harris et al. [33] evaluated different ML models using the Concordance-statistic (C-statistic), which shows the probability of a model successfully differentiating between two outcomes (e.g., post-operative PROM improvement versus no improvement), with C > 0.5 showing meaningful predictive value and C = 1.0 indicating perfect prediction. They reported values between 0.72 and 0.76 for least absolute shrinkage and selection operator (LASSO) regression, gradient boosting, and quadratic discriminant analysis across different Knee Injury and Osteoarthritis Outcome Score (KOOS) scales. Huber et al. [32] reported areas under the curve (AUCs) ranging from 0.87 to 0.86 for a Visual Analogue Scale (VAS), and 0.78 for a hip arthroplasty Q-score, with 0.71 for a knee arthroplasty Q-score. The top-performing algorithms were XGB, neural nets, multi-step elastic net, and generalized linear models.

Hoogendam et al. [30] found that gradient boosting (AUC = 0.782) and generalized linear models (AUC = 0.781) exhibited similar accuracy when predicting postoperative PROMs, while RFs had a significantly lower AUC (0.735, *p* = 0.017 compared to gradient boosting). Kumar et al. [31] compared linear regression, XGB, and Wide & Deep networks, finding that the Wide & Deep model had the smallest mean absolute error for predicting postoperative PROM scores. Zhou et al. [29] compared a traditional logistic regression model (AUC = 0.712) with three ML models: classification tree (AUC = 0.657), XGB (AUC = 0.676), and RF (AUC = 0.671)—and found logistic regression to be superior. Lötsch et al. [34] used an RF algorithm to select items from different non-CAT PROMs, effectively predicting the development of persistent pain after breast cancer surgery. Their ML model combined items from two different PROMs and provided equal predictive accuracy as the full PROMs together. The final 7-item model exhibited a sensitivity of 0.62, specificity of 0.65, and balanced accuracy of 0.63, comparable to the combined accuracy of the four individual full-length PROMs, totaling 73 items, used in the study (0.56 to 0.63). Similarly, Campagner et al. [35] used multiple ML algorithms to develop a model identifying patients suitable for fast-track surgical protocols. They concluded that all models produced good-to-excellent sensitivity and specificity, with AUCs ranging from 0.77 to 0.85 across eight different ML algorithms.

### Impact of AI and ML on patient questionnaire burden

Three studies evaluated the reduction in the number of items/questions required for CATs compared to the full-length PROMs. Banerjee et al. [19] reported a 30% reduction in items for the CAT version of the Hip Disability and Osteoarthritis Outcome Score (HOOS), which used 28 items compared to 40 in the full-length HOOS. Young Afat et al. [20] demonstrated a reduction of 37.5% in items between the BREAST-Q PROM and the CAT version when the standard error was set at an acceptable level for individual measurement, with the CAT using 10 items compared to 16 in the full-length version. This reduction increased to 75% (4 items) for group-based measurements (e.g., for use in a clinical trial where PROM results are compared at the intervention group level). Similarly, the CAT version of the CLEFT-Q reduced the number of items from 76 to 59, achieving a 22.4% reduction, with the accuracy remaining the same. Additionally, one study utilized ML techniques to reduce questionnaire length by combining items from different PROMs into a single predictive score. Lötsch et al. [34] successfully condensed 73 original items into a 7-item set, achieving a 90.4% reduction, while still accurately predicting persistent post-surgical pain.

## Discussion

To our knowledge, this is the first systematic review to specifically evaluate AI- and ML-based methods for PROM development, application, and outcome prediction in surgical populations. While AI in healthcare encompasses many technologies, CATs are among the most widely implemented AI-enabled methods in PROM measurement, particularly in surgery, due to their ability to reduce patient burden while maintaining psychometric rigor. Nevertheless, these measures are relevant beyond surgery [36]. Additionally, ML-based models are of pivotal importance for outcome prediction [37]. Consequently, this review has demonstrated that AI and ML can significantly enhance the use of PROMs in surgical practice.

Three studies showed that CAT PROMs closely align with those of their full-length counterparts, exhibiting near-perfect agreement [16, 19, 20]. The psychometric properties of CAT PROMs, particularly measurement error and construct validity, were consistently rated as good to excellent. Although some studies reported slightly elevated floor and ceiling effects compared to traditional PROMs, these were generally within acceptable limits, with only one exception noted. These findings offer interesting perspectives, and CAT PROMs can be used in both clinical practice and research contexts to construct other PROM scores. Additionally, all studies investigating patient questionnaire burden reported substantial reductions in the number of items required, with a reduction rate ranging from 22% to 90%.

The review also highlighted the successful application of ML algorithms in constructing prediction models for post-surgical PROM outcomes. However, there was no consistent evidence supporting the superiority of any specific ML algorithm. Minor differences in model performance were observed across studies, with various algorithms showing the best performance in different contexts. Notably, Zhou et al. [29] found that advanced ML models did not always outperform traditional statistical methods, such as logistic regression, in predicting PROM outcomes. The studies included in this review represent pioneering efforts in leveraging AI and ML to track PROMs post-surgery. The rise of AI and ML is expected to play a transformative role in managing the increasingly vast amounts of data being produced in healthcare. Furthermore, as healthcare systems increasingly prioritize patient-centered care, PROMs are becoming essential for capturing patient experiences and outcomes [38].

However, the challenge lies in analyzing and utilizing this data in real-time, given constraints on resources such as clinician time and digital infrastructure [39]. AI and ML have the potential to address these challenges by processing extensive data from PROMs alongside clinical data such as biomarkers, imaging, and inputs from wearable sensor data on heart rate and biomechanics, to enable personalized treatment and recovery plans. By integrating PROMs with other clinical factors, AI models can predict treatment outcomes, identify candidates for fast-track surgery (i.e., enhanced recovery pathways designed to minimize hospital stay and accelerate postoperative recovery), and determine which interventions are most likely to improve quality of life for individual patients. This capability enhances the precision of care, leading to better clinical outcomes and increased patient satisfaction [40, 41]​.

Moreover, AI-driven models have the potential to continuously analyze PROMs over time, offering dynamic, real-time insights into patients’ progress and perceptions of their health outcomes. This capability can significantly enhance shared decision-making between clinicians and patients [42]. For instance, AI systems can identify patterns in PROMs to alert healthcare providers to declining patient-reported well-being, enabling early interventions to prevent complications or hospital readmissions [41]. These models can also optimize care delivery, with studies showing that ML-powered systems improve both patient outcomes and satisfaction by more accurately predicting recovery trajectories and adjusting care plans accordingly [43].

As the volume and complexity of patient-reported data increase, AI and ML technologies are expected to become indispensable for enhancing the quality and personalization of medical care [44]. Interestingly, the studies included in this review on ML-based prediction modeling did not consistently demonstrate the superiority of any specific algorithm. This lack of clear differentiation is likely due to the nature of the data being analyzed and the shared statistical principles underlying these models. Algorithms such as logistic regression, RFs, and neural networks are all capable of detecting relationships between variables when trained on high-quality, well-curated datasets. Despite differences in complexity, these algorithms often converge on similar predictive capabilities because they optimize comparable objective functions, such as minimizing error or maximizing accuracy [45]. In healthcare, where variables and outcomes are typically well-defined, a range of algorithms can identify the same key predictors, especially when modeling is based solely on PROM data, as was the case in this review. For example, studies comparing support vector machines, decision trees, and neural networks have shown that with appropriate hyperparameter tuning and cross-validation, these algorithms converge on similar predictive capabilities because they optimize comparable objective functions in predicting complications or recovery times after surgery [46]. This performance parity underscores the importance of data quality and engineering over algorithm choice in predictive modeling for PROM outcomes. It is interesting to note that the majority of the included studies were on orthopedic and plastic surgery populations. This may be partly due to the nature of these specialties, where surgical outcomes often rely heavily on patients’ self-reported experiences rather than a particular objective measure.

This review has some limitations. In focusing solely on models using PROM data for prediction, we excluded different studies that included a wide variety of clinical factors in their modelling. While this narrow scope may limit the generalizability of our findings regarding the equivalence of ML algorithms, we are confident that the result holds due to the inherent nature of these algorithms, as discussed above. Another limitation is that the use of CAT PROMs in surgical populations is still relatively limited, leaving several questions unanswered. Key issues include digital exclusion, digital literacy, and the availability of computer and internet infrastructures in healthcare facilities worldwide. Nevertheless, in populations and settings with high digital literacy and experience, our conclusion that CAT PROMs can simplify data collection and reduce patient burden without compromising data quality remains valid. Despite limitations, from our perspective, the value of combining different angles (AI-based measurement efficiency and outcome prediction from ML) is useful to explain a continuum between how PROM data are collected (CATs) and how they are used (ML-based prediction). This approach could allow for deeper methodological detail (e.g., statistics-focused analyses) to better dissect the phenomenon. Additionally, the predictive capacity of PROMs in surgical populations is clinically relevant for planning and tailoring postoperative care. Future research might consider separating these domains to provide clearer guidance on either the application of AI/ML methods or the prediction of post-surgical outcomes.

## Conclusions

This systematic review demonstrates that AI and ML have the potential to improve the use of PROMs in surgical care, thereby advancing patient-centered and personalized healthcare. CAT PROMs exhibit psychometric properties comparable to full-length traditional PROMs while significantly reducing patient burden. Furthermore, ML algorithms enable the integration of data from multiple PROMs to calculate key indices of post-surgical recovery and accurately predict outcomes for health domains that are of critical importance to patients. Finally, given the positive results of AI/ML approaches in surgical PROM contexts, similar systematic investigations in pharmacological intervention studies and other contexts, could provide valuable insights into patient-centered outcomes, particularly in chronic disease management where PROMs play a central role.

## Supplementary Information

Below is the link to the electronic supplementary material.

Supplementary Material 1

## Data Availability

All relevant data were presented within the manuscript.
