# Supplementary material for: Machine learning in the development and application of patient-reported outcome measures (PROMs) for surgical patients: a systematic review
Source: J Patient Rep Outcomes. 2026 Jan 14;10:20. doi: 10.1186/s41687-026-00992-8 (PMC12891313; doi:10.1186/s41687-026-00992-8)
Supplement: Supplementary file 1 — Supplementary Material 1 [file 41687_2026_992_MOESM1_ESM.docx]

**Supplemental Online Content**

**eMethods**

**eFigure.** PRISMA flow chart summarizing the article screening workflow.

**eMethods**

Full PubMed search syntax.

(("computer*"[All Fields] AND "adj2"[All Fields] AND ("acclimatization"[MeSH Terms] OR "acclimatization"[All Fields] OR "adaptation"[All Fields] OR "adaptations"[All Fields] OR "adapt"[All Fields] OR "adaptabilities"[All Fields] OR "adaptability"[All Fields] OR "adaptable"[All Fields] OR "adaptational"[All Fields] OR "adaptative"[All Fields] OR "adapte"[All Fields] OR "adapted"[All Fields] OR "adapting"[All Fields] OR "adaption"[All Fields] OR "adaptions"[All Fields] OR "adaptive"[All Fields] OR "adaptively"[All Fields] OR "adaptiveness"[All Fields] OR "adaptivity"[All Fields] OR "adapts"[All Fields])) OR ("algorithm s"[All Fields] OR "algorithmic"[All Fields] OR "algorithmically"[All Fields] OR "algorithmics"[All Fields] OR "algorithmization"[All Fields] OR "algorithms"[MeSH Terms] OR "algorithms"[All Fields] OR "algorithm"[All Fields]) OR "Item bank"[All Fields])

AND ("psychometrics"[MeSH Terms] OR "psychometr*"[Title/Abstract] OR "clinimetr*"[Text Word] OR "clinometr*"[Text Word] OR "outcome assessment"[Title/Abstract] OR "outcome measure*"[Text Word] OR "Health Status Indicators"[MeSH Terms] OR "reproducibility of results"[MeSH Terms] OR "reproducib*"[Title/Abstract] OR "discriminant analysis"[MeSH Terms] OR "reliab*"[Title/Abstract] OR "unreliab*"[Title/Abstract] OR "valid*"[Title/Abstract] OR "coefficient of variation"[Title/Abstract] OR "coefficient"[Title/Abstract] OR "homogeneity"[Title/Abstract] OR "homogeneous"[Title/Abstract] OR "internal consistency"[Title/Abstract] OR ("cronbach*"[Title/Abstract] AND ("alpha"[Title/Abstract] OR "alphas"[Title/Abstract])) OR ("item"[Title/Abstract] AND ("correlation*"[Title/Abstract] OR "selection*"[Title/Abstract] OR "reduction*"[Title/Abstract])) OR "agreement"[Text Word] OR "precision"[Text Word] OR "imprecision"[Text Word] OR "precise values"[Text Word] OR "test-retest"[Title/Abstract] OR ("test"[Title/Abstract] AND "retest"[Title/Abstract]) OR ("reliab*"[Title/Abstract] AND ("test"[Title/Abstract] OR "retest"[Title/Abstract])) OR "stability"[Title/Abstract] OR "interindividual"[Title/Abstract] OR "inter-individual"[Title/Abstract] OR "intraindividual"[Title/Abstract] OR "intra-individual"[Title/Abstract] OR "interparticipant"[Title/Abstract] OR "inter-participant"[Title/Abstract] OR "intraparticipant"[Title/Abstract] OR "intra-participant"[Title/Abstract] OR "kappa"[Title/Abstract] OR "kappa's"[Title/Abstract] OR "kappas"[Title/Abstract] OR "repeatab*"[Text Word] OR (("replicab*"[Text Word] OR "repeated"[Text Word]) AND ("measure"[Text Word] OR "measures"[Text Word] OR "findings"[Text Word] OR "result"[Text Word] OR "results"[Text Word] OR "test"[Text Word] OR "tests"[Text Word])) OR "generaliza*"[Title/Abstract] OR "generalisa*"[Title/Abstract] OR "concordance"[Title/Abstract] OR ("intraclass"[Title/Abstract] AND "correlation*"[Title/Abstract]) OR "discriminative"[Title/Abstract] OR "known group"[Title/Abstract] OR "factor analysis"[Title/Abstract] OR "factor analyses"[Title/Abstract] OR "factor structure"[Title/Abstract] OR "factor structures"[Title/Abstract] OR "dimension*"[Title/Abstract] OR "subscale*"[Title/Abstract] OR ("multitrait"[Title/Abstract] AND "scaling"[Title/Abstract] AND ("analysis"[Title/Abstract] OR "analyses"[Title/Abstract])) OR "item discriminant"[Title/Abstract] OR "interscale correlation*"[Title/Abstract] OR "error"[Title/Abstract] OR "errors"[Title/Abstract] OR "individual variability"[Title/Abstract] OR "interval variability"[Title/Abstract] OR ("variability"[Title/Abstract] AND ("analysis"[Title/Abstract] OR "values"[Title/Abstract])) OR ("uncertainty"[Title/Abstract] AND ("measurement"[Title/Abstract] OR "measuring"[Title/Abstract])) OR "standard error of measurement"[Title/Abstract] OR "sensitiv*"[Title/Abstract] OR "responsive*"[Title/Abstract] OR ("limit"[Title/Abstract] AND "detection"[Title/Abstract]) OR "interpretab*"[Title/Abstract] OR (("minimal"[Title/Abstract] OR "minimally"[Title/Abstract] OR "clinical"[Title/Abstract] OR "clinically"[Title/Abstract]) AND ("important"[Title/Abstract] OR "detectable"[Title/Abstract]) AND ("change"[Title/Abstract] OR "difference"[Title/Abstract])) OR ("small*"[Title/Abstract] AND ("real"[Title/Abstract] OR "detectable"[Title/Abstract]) AND ("change"[Title/Abstract] OR "difference"[Title/Abstract])) OR "meaningful change"[Title/Abstract] OR "ceiling effect"[Title/Abstract] OR "floor effect"[Title/Abstract] OR "Item response model"[Title/Abstract] OR "IRT"[Title/Abstract] OR "Rasch"[Title/Abstract] OR "Differential item functioning"[Title/Abstract] OR "DIF"[Title/Abstract] OR "computer adaptive testing"[Title/Abstract] OR "Item bank"[Title/Abstract] OR "cross-cultural equivalence"[Title/Abstract])

AND ("PROM"[Title/Abstract] OR "patient report*"[Title/Abstract] OR "self report*"[Title/Abstract] OR "questionnaire"[Title/Abstract] OR "survey"[Title/Abstract] OR "scale*"[Title/Abstract] OR "user"[Title/Abstract] OR "accept*"[Title/Abstract] OR "feasib*"[Title/Abstract])

AND ("surgery"[MeSH Subheading] OR "surgery"[All Fields] OR "surgical procedures, operative"[MeSH Terms] OR ("surgical"[All Fields] AND "procedures"[All Fields] AND "operative"[All Fields]) OR "operative surgical procedures"[All Fields] OR "general surgery"[MeSH Terms] OR ("general"[All Fields] AND "surgery"[All Fields]) OR "general surgery"[All Fields] OR "surgery s"[All Fields] OR "surgerys"[All Fields] OR "surgeries"[All Fields] OR "operation"[Title/Abstract] OR "operative"[Title/Abstract])) NOT (("review"[Publication Type] OR "review literature as topic"[MeSH Terms] OR "review"[All Fields]) OR robot*)

**eFigure.** PRISMA flow chart summarizing the article screening workflow.

**Identification of studies**

Records removed *before screening*:

n = 0

Records identified from PubMed search:

n = 6,463

**Identification**

Records screened

(n = 6,463)

Records excluded**

(n = 6,432)

Reports sought for retrieval

(n = 31)

Reports not retrieved

(n = 0)

**Screening**

Reports excluded:

Non-surgical population (n = 1)

Not about PROMs (n = 4)

No AI involvement (n = 4)

Reports assessed for eligibility

(n = 31)

Studies included in review

(n = 22)

**Included**
